# Lysophospholipids in Synucleinopathies: A Conceptual Framework Linking Proteostasis and Neuroinflammatory Signaling

**DOI:** 10.3390/brainsci16050485

**Published:** 2026-04-30

**Authors:** Tamotsu Tsukahara, Hisao Haniu, Yoshikazu Matsuda

**Affiliations:** 1 Division of Clinical Pharmacology and Pharmaceutics, Nihon Pharmaceutical University, Ina 362-0806, Japan; yomatsuda@nichiyaku.ac.jp; 2 SLC Pharmaceutical Co., Ltd., Tokyo 107-0052, Japan; 3 Institute for Biomedical Sciences, Research Cluster for Social Implementation, Shinshu University, Matsumoto 390-8621, Japan; hhaniu@shinshu-u.ac.jp

**Keywords:** α-synuclein, lysophospholipids, neuroinflammation, proteostasis, microglia, mitochondrial dysfunction, neurodegenerative diseases, synucleinopathies, gut–brain axis

## Abstract

**Highlights:**

**What are the main findings?**

- Lysophospholipids are key regulators linking neuroinflammation and proteostasis in synucleinopathies.
- Lipid-mediated mechanisms may influence α-Syn aggregation and disease progression.

**What are the implications of the main findings?**

- Targeting lipid signaling pathways may offer a novel strategy for modulating synucleinopathies.
- A lipid-centric framework may help integrate diverse pathological processes in neurodegenerative diseases.

**Abstract:**

Synucleinopathies, including Parkinson’s disease and dementia with Lewy bodies, are characterized by progressive α-synuclein (α-Syn) aggregation accompanied by chronic neuroinflammatory changes. However, the mechanistic relationship between disrupted proteostasis and inflammatory signaling remains incompletely defined and may vary across disease stages and clinical subtypes. Lysophospholipids (LPLs) are bioactive lipids derived from membrane phospholipids that participate in diverse cellular processes. These functions are primarily mediated through G protein-coupled receptor (GPCR) signaling, but may also involve direct effects on membrane organization and biophysical properties. In addition to receptor-mediated pathways, the surrounding lipid environment may influence protein behavior, although its role in neurodegenerative processes remains to be fully elucidated. Within this framework, LPLs can be considered not only as signaling molecules but also as modulators of the cellular environment in which proteostasis and inflammatory responses occur. In this review, we adopt a lipid-centered perspective in which LPLs occupy an interface between lipid signaling, protein aggregation, and neuroinflammation. Rather than acting as a single initiating factor, altered lipid metabolism is likely to contribute through multiple interconnected pathways. Although current evidence is largely derived from preclinical studies, it supports a role for lipid-related mechanisms, particularly in early stages of synucleinopathy.

## 1. Introduction

**Figure 1:**
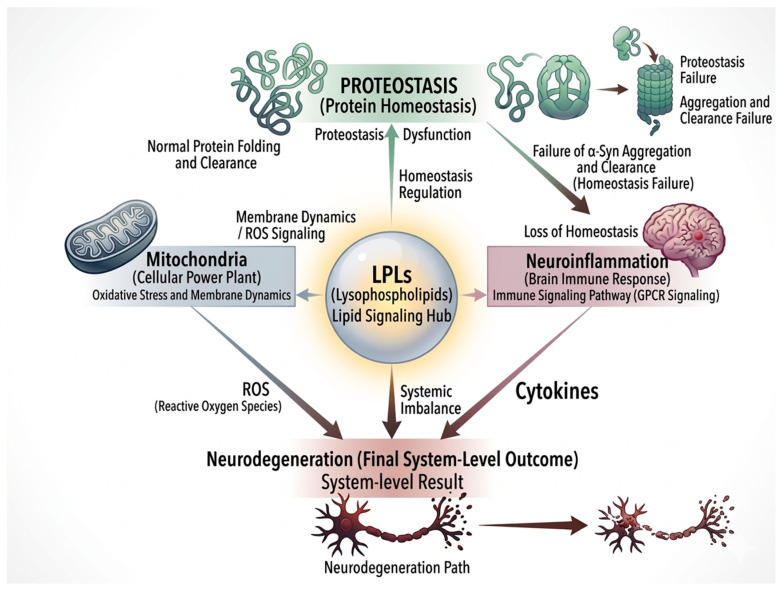
Proposed lysophospholipid-centered regulatory network in synucleinopathies. LPLs are proposed to modulate proteostasis, mitochondrial function, and neuroinflammation in synucleinopathies. In this hypothetical model, LPLs may influence α-Syn aggregation and clearance, affect mitochondrial homeostasis via oxidative stress and membrane dynamics, and regulate neuroinflammatory signaling through GPCR-dependent pathways. These processes may form an interconnected network with potential feed-forward interactions; however, the exact relationships remain context-dependent and incompletely defined.

Recently, neuroinflammation and neurodegeneration are increasingly recognized as common features across a range of neuropsychiatric and neurodegenerative disorders, although their roles vary depending on disease context and stage [1,2]. In many cases, sustained microglial activation is linked with the release of inflammatory mediators and reactive oxygen species (ROS), and these processes have been implicated in neuronal dysfunction and synaptic alterations [3,4]. These changes are often discussed in relation to diseases with abnormal α-Syn aggregation, such as Parkinson’s disease (PD), dementia with Lewy bodies (DLB), and multiple system atrophy (MSA) [5,6]. In these disorders, misfolded α-Syn builds up in neurons and also in glial cells, forming inclusions like Lewy bodies and Lewy neurites [7,8]. While these pathological features are well established, their direct contribution to neurodegenerative processes remains incompletely understood. This uncertainty has prompted increasing interest in the interplay between protein aggregation, cellular stress responses, and neuroinflammatory signaling. Within this context, lipid-related mechanisms have emerged as potential regulatory factors, although their roles are not yet fully defined. Phospholipids and LPLs, traditionally considered structural components of cellular membranes, are now recognized as bioactive molecules involved in signaling pathways, mitochondrial function, and synaptic regulation [9,10]. Alterations in lipid metabolism have been reported in multiple neurodegenerative disorders, including synucleinopathies, although their functional significance remains unclear [11]. Porcine liver decomposition product (PLDP) represents one example of a multi-component lipid-containing system. LP it is a mixture produced by enzymatic hydrolysis of porcine liver and contains phospholipids, lysophospholipids, peptides, and amino acids [12]. Several experimental studies [13,14,15,16,17] have suggested that PLDP may influence microglial activity and neuroinflammatory responses; however, these findings are primarily derived from preclinical models and remain to be validated in human systems. However, due to its complex composition, the specific components responsible for these effects remain difficult to identify. More broadly, these observations raise the question of whether lipid-related processes may contribute more directly to protein homeostasis than traditionally assumed. Among lipid species, lysophospholipids are of particular interest because of their potential to influence membrane properties and protein–lipid interactions. Although emerging evidence suggests that LPLs may affect early stages of protein misfolding or aggregation, current findings remain limited and context-dependent. In this review, we propose a conceptual framework in which lysophospholipids are considered modulators of membrane-associated processes linking proteostasis and neuroinflammatory signaling. Within this framework, lipid signaling is integrated with proteostasis and inflammation rather than viewed as a downstream consequence. Multi-component lipid systems, including PLDP, are discussed as illustrative examples, although their precise roles remain to be fully clarified. Most available evidence is derived from experimental models, and its relevance to human disease remains to be established. Finally, lysophosphatidylcholine (LPC), a major component of PLDP, is highlighted as a lipid of interest; however, its role in α-Syn aggregation and inflammatory signaling remains under investigation. In this review, LPLs are discussed as a broad class of bioactive lipids, while LPC is treated as a representative but not exclusive molecular species. PLDP is considered a multicomponent preparation that may influence LPL-related pathways but should not be equated with individual LPL species (see Figure 1). This distinction is maintained throughout the manuscript.

## 2. LPLs in Inflammatory Diseases in Central Nervous System

### 2.1. Molecular Mechanisms and Pathophysiological Roles

LPC is a class of bioactive lipids generated from membrane phospholipids through the action of phospholipase A_2_ and has been increasingly recognized as a potential link between lipid metabolism and inflammatory processes [18]. Current research has primarily focused on its involvement in G protein-coupled receptor (GPCR) signaling pathways, including lysophosphatidic acid receptors (LPA_1_–LPA_6_) and sphingosine-1-phosphate receptors (S1P_1_–S1P_5_) [19,20,21]. These receptors engage multiple downstream cascades—Rho/ROCK, PI3K/Akt, MAPK/ERK, and PLC/Ca^2+^ signaling, among others—that ultimately regulate cytoskeletal organization and cell survival [22]. Although this receptor-mediated framework is well established, it may not fully account for the broader functional roles of LPLs within complex lipid environments. In addition to receptor-dependent signaling, the amphiphilic nature of LPLs suggests that they can directly influence membrane properties [23]. Alterations in membrane curvature, fluidity, and microdomain organization may affect the formation and stability of signaling complexes, potentially modulating cellular responses independently of ligand–receptor interactions. Alterations in membrane curvature, fluidity, and microdomain organization may affect the formation and stability of signaling complexes, potentially modulating cellular responses independently of ligand–receptor interactions. These biophysical effects are often difficult to distinguish experimentally from canonical signaling pathways, and may operate concurrently within cellular systems. Furthermore, LPLs may interact with intracellular or membrane-associated proteins. For example, α-Syn has been shown to be sensitive to its lipid environment, although the underlying mechanisms remain incompletely defined and may vary across experimental models. Changes in lipid composition have been proposed to influence early stages of protein aggregation; however, current evidence remains limited and does not yet support a unified mechanistic model. The regulation of LPL biology is further controlled by enzymatic and transport systems. Enzymes such as autotaxin (ATX) and lipid phosphate phosphatases (LPPs) tightly control LPL production and degradation [21], while transport systems determine when and where these molecules become available. MFSD2A-mediated transport across the blood–brain barrier (BBB) represents one well-characterized pathway [22], although additional transport mechanisms are likely to exist but remain less well defined. The BBB is increasingly recognized as a dynamic and functionally heterogeneous interface rather than a strictly binary structure. In addition to changes in permeability, alterations in transport activity, endothelial signaling, and inflammatory responsiveness may contribute to disease-related processes. These mechanisms may facilitate interactions between peripheral factors and the central nervous system, potentially including misfolded α-Syn species [24,25,26,27]. Although the extent to which such interactions contribute to disease progression remains unclear, they support a model of active peripheral–central communication. Among LPLs, LPC has received particular attention due to its relatively high abundance in circulation and its association with endothelial and barrier-related responses. Alterations in LPC levels have been associated with endothelial activation and inflammatory signaling, including NF-κB pathway activation and upregulation of adhesion molecules such as ICAM-1 and VCAM-1 [28]. These effects may influence BBB function, although the magnitude and direction of responses appear to depend on experimental and pathological context [29]. Clinical lipidomic studies have reported altered LPC profiles in Parkinson’s disease; however, the relationship between peripheral lipid changes and central nervous system pathology remains incompletely understood. Nevertheless, these observations suggest that peripheral lipid metabolism and brain pathology are functionally interconnected. Taken together, LPC and related LPL mechanisms—particularly those involving endothelial function and BBB dynamics—can be considered modulators of synuclein-associated pathology rather than primary drivers. Their contributions may vary depending on disease stage and context, and further studies are required to clarify their precise roles.

### 2.2. LPLs as Integrators of Neuroinflammation and Therapeutic Targets

Building on these molecular and neurovascular observations, particularly those related to LPL-associated BBB dysfunction, lysophospholipids may be considered potential integrators of neuroinflammatory signaling in the central nervous system. This role may be particularly relevant in microglia, which are highly responsive to changes in the neurovascular environment. In these cells, LPL-related GPCR signaling has been reported to engage pathways such as NF-κB and MAPK, leading to the production of pro-inflammatory cytokines and chemokines [12,30]. In this context, LPLs may function upstream of innate immune activation, although their effects are likely to be context-dependent. Their involvement is not restricted to a single stage of the inflammatory response. Emerging evidence suggests that LPL signaling may participate in both priming and activation phases of inflammasome-related pathways [31]. However, the mechanisms coordinating these processes remain incompletely understood. One proposed mechanism is that receptor-mediated signaling and membrane remodeling jointly contribute to NLRP3 inflammasome assembly under specific pathological conditions [32]. Although this hypothesis remains to be fully validated, it provides a conceptual link between membrane dynamics and inflammatory amplification. An additional consideration is the potential interaction between these pathways and proteostasis. Because LPLs influence membrane organization and protein–lipid interactions, they may indirectly affect processes related to protein misfolding and aggregation. This is particularly relevant in synucleinopathies, where α-Syn aggregation is a defining pathological feature. However, distinguishing direct lipid–protein interactions from secondary effects related to altered cellular states remains challenging. Taken together, these observations suggest that LPLs operate across multiple levels of regulation rather than within a single linear pathway. Their involvement in both inflammatory signaling and proteostasis supports a model in which LPLs act as integrative modulators, although the precise boundaries of this role remain to be defined. From a therapeutic perspective, this complexity presents both opportunities and challenges. Several components of the LPL signaling system—including LPA/S1P receptors, autotaxin (ATX), and lipid transport at the BBB—are theoretically druggable targets [33,34]. However, broad inhibition of these pathways may interfere with essential physiological functions. Therefore, therapeutic strategies are likely to require context-dependent or partial modulation rather than complete inhibition. Overall, LPLs may represent a point of convergence between lipid metabolism, immune signaling, and protein homeostasis. Whether these processes constitute primary drivers of pathology or secondary responses remains unclear. At present, this framework provides a useful basis for integrating multiple aspects of neurodegenerative disease mechanisms.

## 3. Neuroinflammatory Mechanisms in Synucleinopathies

### 3.1. Lysophospholipids in α-Syn Proteostasis

**Figure 2:**
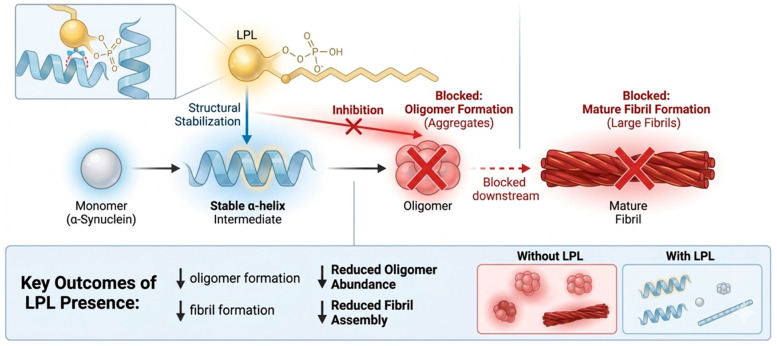
Proposed mechanism of lysophospholipid-mediated modulation of α-Syn aggregation. LPLs are proposed to influence the conformational dynamics of α-Syn. In the absence of LPLs, α-Syn monomers may undergo misfolding and form oligomeric and fibrillar species associated with neurotoxicity. In contrast, LPLs may promote stabilization of α-helical intermediates, potentially reducing oligomerization and fibril formation. However, these effects remain mechanistic hypotheses and require further experimental validation.

Synucleinopathies are a group of proteinopathies, including Parkinson’s disease (PD), dementia with Lewy bodies (DLB), and multiple system atrophy (MSA), characterized by abnormal α-Syn accumulation and aggregation, which is associated with neuroinflammatory responses [6]. In particular, soluble oligomeric forms of α-Syn are widely considered to exhibit increased neurotoxicity and can be released into the extracellular space, where they may act as damage-associated molecular patterns (DAMPs) [35]. These α-Syn species are recognized by pattern recognition receptors, including Toll-like receptors (TLR2 and TLR4), thereby triggering downstream inflammatory signaling cascades [36]. At the molecular level, α-Syn aggregation is thought to involve electrostatic interactions and to proceed through oligomeric intermediates, which are often regarded as among the most cytotoxic species [37]. However, the relationship between aggregation state and toxicity remains incompletely defined. Structurally, α-Syn contains an N-terminal membrane-binding region, a hydrophobic core that drives fibril formation, and a negatively charged C-terminal domain [38]. Post-translational modifications, including phosphorylation and ubiquitination, modulate aggregation propensity, although their relative contributions likely depend on cellular context and disease stage. Increasing attention has recently been directed toward the role of lipid environments in regulating these processes. LPLs, in particular, have been proposed to influence α-Syn aggregation through both indirect mechanisms and potential direct molecular interactions. Several studies—including our previous work [13,39]—have reported that specific LPL species can modulate α-Syn aggregation in vitro and in cellular models, suggesting a role of lipid composition in α-Syn misfolding dynamics. For instance, LPL fractions derived from PLDP, a lipid-rich extract, have been reported to reduce β-sheet-rich fibril formation in ThT-based assays and to attenuate intracellular aggregation and cytotoxicity in neuronal systems [13,39,40,41]. These observations suggest that LPLs may influence early aggregation processes, including nucleation and oligomer formation (see Figure 2), although this remains to be fully established. One proposed mechanism relates to the amphiphilic nature of LPLs. The N-terminal region of α-Syn contains amphipathic motifs that mediate interactions with lipid membranes and partially folded intermediates [42]. LPLs, which possess both a polar head group and a single acyl chain, may interact with these regions through hydrophobic and electrostatic interactions [43]. Such interactions could stabilize non-aggregating conformations and reduce the probability of oligomer formation, although direct structural evidence remains limited. This potential mechanism differs from that of classical protein chaperones. Unlike large protein complexes, LPLs are small and diffusible molecules that may act across both membrane-associated and extracellular compartments. This suggests that lipid-mediated modulation may not be restricted to a single step in the α-Syn lifecycle but could occur at multiple stages. However, the extent and physiological relevance of these effects remain to be clarified. At present, much of the available evidence is indirect. The specificity of LPL–α-Syn interactions, as well as their binding modes and in vivo relevance, are not yet fully defined. Experimental observations, including those from our studies, indicate that the magnitude of these effects may vary depending on lipid composition and experimental conditions, highlighting the importance of local biochemical context. Taken together, these findings support a model in which LPLs function as modulators of α-Syn proteostasis, particularly during early stages of aggregation. Within this framework, lipid–protein interactions represent a regulatory interface linking membrane biology and protein homeostasis. Importantly, these effects are likely to be context-dependent and may vary with lipid composition, concentration, and cellular state. Accordingly, LPLs may exert both protective and, under certain conditions, potentially deleterious effects.

### 3.2. Microglial Activation and Pro-Inflammatory Signaling

LPLs have been proposed to modulate microglial activation and neuroinflammatory responses through receptor-mediated signaling pathways. Microglia are the main innate immune cells in the central nervous system (CNS) and continuously monitor their local environment [3,44]. In synucleinopathies, misfolded α-Syn—particularly in its soluble oligomeric forms—is widely regarded as a potent trigger of microglial activation, acting as a damage-associated molecular pattern (DAMP) [45]. These species are sensed by several pattern recognition receptors (PRRs), including TLR2, TLR4, CD36, and RAGE, which then initiate downstream inflammatory signaling pathways [35,46]. Once these receptors are engaged, adaptor proteins such as MyD88 activate intracellular cascades involving NF-κB and MAPK pathways (ERK, JNK, and p38), leading to the production of pro-inflammatory cytokines (e.g., TNF-α, IL-1β, IL-6) and chemokines such as CCL2 [36,47,48]. Induction of enzymes such as iNOS and COX-2 further contributes to the inflammatory response. These signaling pathways are relatively well characterized at the molecular level. In parallel, internalized α-Syn can trigger intracellular stress responses that contribute to activation of the NLRP3 inflammasome [49]. This process is thought to involve lysosomal disruption and mitochondrial dysfunction, resulting in the generation of signals such as reactive oxygen species (ROS) and cathepsin release. These eventually converge on caspase-1 activation and maturation of IL-1β and IL-18 [50]. However, this framework is likely to be more complex than a simple linear cascade. Microglial activation in vivo is influenced by multiple interacting factors, including the lipid microenvironment. Alterations in membrane composition, particularly within lipid raft domains, can affect receptor organization and signaling efficiency [51]. Although these effects are challenging to isolate experimentally, accumulating evidence supports their functional relevance. LPLs may contribute to this regulatory layer. Rather than acting solely as signaling ligands, LPLs may modulate the activation threshold of microglia through combined effects on membrane organization and receptor signaling. Some studies report effects on NF-κB signaling and inflammasome-related pathways [52,53], although findings are not entirely consistent across experimental systems. These discrepancies likely reflect the context-dependent nature of LPL signaling, including differences in cell type, lipid composition, and stimulus conditions. Metabolic reprogramming represents an additional regulatory dimension. Activated microglia generally shift toward glycolysis, with HIF-1α and mTOR involved in this transition [54]. Chronic exposure to α-Syn has been associated with reduced phagocytic capacity, potentially impairing the clearance of aggregated proteins. This may contribute to a self-reinforcing cycle between inflammation and protein accumulation. Activated microglia also produce ROS and reactive nitrogen species through NOX2 and iNOS, which can be harmful to surrounding neurons [55]. In addition, microglia-derived cytokines influence astrocytes and endothelial cells, promoting astrocytic reactivity and potentially altering BBB function. These changes may facilitate the entry of peripheral immune signals or cells into the CNS, although their contribution to disease progression remains incompletely defined [56]. Taken together, microglial activation in synucleinopathies is best understood as a network of interacting processes rather than a strictly linear pathway. Receptor signaling, intracellular stress responses, metabolic reprogramming, and intercellular communication are likely to act in a coordinated and context-dependent manner. Within this framework, LPLs—particularly LPC—can be considered modulators rather than primary drivers of microglial activation [57]. By influencing membrane organization and GPCR signaling, they may regulate NF-κB activation and inflammasome priming. However, the direction and magnitude of these effects are likely to vary depending on experimental and pathological context.

### 3.3. Astrocyte Reactivity and Neuroinflammatory Amplification

LPLs have also been implicated in the regulation of astrocytic functions, potentially influencing inflammatory signaling and metabolic support within the central nervous system. Astrocytes are abundant glial cells in the central nervous system (CNS) that perform diverse functions in health and disease, supporting homeostatic processes including neurotransmitter clearance, ion buffering, metabolic support, and maintenance of BBB integrity [58,59]. Under physiological conditions, these functions are tightly regulated. In synucleinopathies, however, this homeostatic balance is frequently disrupted. In disorders such as Parkinson’s disease and related conditions, astrocytes are exposed to multiple stressors, including misfolded α-Syn, microglia-derived inflammatory cytokines, and oxidative signals. Their response is commonly referred to as astrogliosis, although this term encompasses heterogeneous and context-dependent states [60]. Rather than representing a single defined phenotype, astrocytes exhibit a spectrum of reactive states. These states are sometimes described using the A1/A2 framework [61]. For example, microglia-derived factors such as TNF-α, IL-1α, and C1q have been reported to induce A1-like astrocytes, which may exhibit reduced homeostatic functions and increased neurotoxic potential [62]. However, this classification is considered an oversimplification, as astrocyte phenotypes in vivo do not consistently conform to discrete categories. At the intracellular level, several signaling pathways are commonly implicated, including NF-κB, JAK/STAT3, and MAPK [63]. These pathways regulate inflammatory gene expression, although their relative contributions vary depending on context. Astrocytes can also take up extracellular α-Syn aggregates. While this process may contribute to clearance, it can also lead to lysosomal dysfunction and sustained activation under pathological conditions [63]. The balance between protective and detrimental effects remains incompletely defined. Astrocytes interact closely with microglia and participate in bidirectional inflammatory signaling. Through the release of cytokines and chemokines, they can modulate microglial activation states, which in turn may influence astrocytic function. This reciprocal interaction may contribute to the persistence of inflammatory responses. Impaired glutamate uptake, potentially due to reduced EAAT1/2 function, may further promote excitotoxicity [64]. Reactive astrocytes have also been reported to produce reactive oxygen and nitrogen species, although the extent of this effect varies across experimental models [65]. Astrocytes also contribute to the regulation of BBB function. Under inflammatory conditions, astrocytes may influence tight junction integrity indirectly and release factors such as VEGF and matrix metalloproteinases (MMPs), which can increase permeability [66]. Although astrocytes are capable of clearing extracellular α-Syn, this capacity may be reduced during disease progression. Metabolic reprogramming, including a shift toward glycolysis, has also been observed, although its causal role remains under investigation. More recently, lipid-related mechanisms have emerged as additional modulators of astrocyte function. Changes in membrane lipid composition may influence astrocyte behavior, although the underlying mechanisms remain to be fully elucidated [67]. Alterations in phospholipids and lysophospholipids can affect membrane properties and the organization of receptor complexes, thereby influencing signaling pathways such as NF-κB and JAK/STAT3 [68]. Lipid microdomains, including raft-like structures, are thought to contribute to these processes, although their specific roles in astrocytes remain incompletely defined. Overall, astrocyte reactivity represents a heterogeneous and dynamic component of neuroinflammatory processes in synucleinopathies. Modulation of inflammatory signaling and membrane lipid dynamics may therefore represent potential therapeutic strategies, although preserving physiological astrocyte functions remains a significant challenge. Within this context, lysophospholipids can be considered modulators of astrocyte reactivity. By influencing membrane organization and signaling platform assembly, they may regulate pathways such as NF-κB and JAK/STAT3 and bias astrocytes toward distinct reactive states. However, these mechanisms remain to be experimentally validated.

### 3.4. Inflammasome Activation and Pyroptosis

Inflammasomes are cytosolic multiprotein complexes that sense cellular stress and initiate inflammatory responses [69]. Among these, the NLRP3 inflammasome has been most extensively studied in the context of synucleinopathies [70], partly because multiple stress signals converge on its activation. The canonical model describes a two-step process consisting of priming and activation [71,72]. Although this framework is widely used, the distinction between these phases is not always clearly defined in complex biological systems. In particular, in chronic conditions, priming and activation processes may occur concurrently or overlap. During priming, expression of NLRP3 and pro-IL-1β increases, typically downstream of receptor-mediated signaling triggered by extracellular stimuli, including misfolded proteins. Activation is associated with intracellular stress signals such as ionic imbalance, mitochondrial dysfunction, and lysosomal damage [73]. However, these processes are not strictly separable and may interact dynamically. In synucleinopathies, α-Syn aggregates can be internalized by microglia and accumulate in lysosomes [74]. Impaired degradation may lead to lysosomal destabilization and release of cathepsins into the cytosol, which has been linked to inflammasome activation [75]. In parallel, mitochondrial dysfunction can result in increased reactive oxygen species (ROS) production and mitochondrial DNA release [76]. These stress signals are often described as distinct triggers but are likely to occur in a coordinated manner. Once NLRP3 is assembled, it recruits ASC and activates caspase-1 [77]. Caspase-1 then processes IL-1β and IL-18 into their mature forms [78], which are released and amplify inflammation [79]. This downstream signaling cascade is relatively well established compared to upstream regulatory mechanisms. Pyroptosis is a recognized downstream consequence of inflammasome activation. Caspase-1 cleaves gasdermin D, producing a pore-forming fragment that disrupts membrane integrity [80]. This process leads to the release of cytokines and intracellular contents and ultimately results in inflammatory cell death [81]. Interactions between inflammasome activation and α-Syn aggregation have also been proposed. Inflammatory signaling may enhance oxidative stress and promote protein misfolding, while impaired clearance mechanisms may allow aggregates to persist [82]. The strength and relevance of this feedback loop are likely to vary depending on experimental and pathological context. Non-canonical inflammasome pathways add further complexity. In humans, caspase-4 and caspase-5 can respond to intracellular lipopolysaccharide and activate gasdermin-dependent pathways independently of NLRP3 [83]. The contribution of these pathways to synucleinopathies remains to be determined. From a therapeutic perspective, multiple components of the inflammasome pathway have been investigated, including NLRP3, caspase-1, and gasdermin D [84]. Upstream processes such as mitochondrial dysfunction and lysosomal stress have also been explored as potential targets [85]. Although these approaches show promise in experimental models, their translational potential remains uncertain. LPLs may represent a modulatory component within this framework. For example, LPC has been reported to influence inflammasome-related signaling in certain experimental contexts [86], potentially through effects on membrane properties and signaling platform organization. However, findings are not fully consistent across studies, suggesting that LPL effects are highly context-dependent. Accordingly, LPLs are more appropriately considered modulators that may influence the sensitivity or threshold of inflammasome activation rather than direct triggers.

### 3.5. Adaptive Immune Responses and Peripheral Immune Involvement

Beyond innate immunity, adaptive immune mechanisms and peripheral immune contributions have increasingly been investigated in synucleinopathies [87]. LPLs have been proposed to modulate adaptive and peripheral immune responses through effects on membrane organization and immune cell signaling, although current evidence remains limited. These processes include antigen presentation, lymphocyte activation, and bidirectional communication between the CNS and peripheral immune compartments, although their in vivo coordination is not yet fully understood. α-Syn has been proposed as a potential antigen source. Microglia—and under certain conditions, peripheral dendritic cells that infiltrate the CNS—can uptake extracellular α-Syn aggregates and process them into peptides [88]. These peptides may be presented via major histocompatibility complex (MHC) class II molecules to CD4^+^ T cells. Cross-presentation via MHC class I pathways, leading to CD8^+^ T cell activation, has also been suggested; however, its relative contribution to disease mechanisms remains unclear [89]. CD4^+^ T cell responses are often characterized by Th1- and Th17-associated cytokine production, including interferon-γ (IFN-γ) and interleukin-17 (IL-17) [90]. These responses are generally associated with pro-inflammatory effects and glial activation, although findings are not fully consistent across studies. Regulatory T cells (Tregs), which normally exert immunosuppressive functions, have been reported to be reduced or functionally impaired in some contexts, although this appears to vary depending on disease stage and experimental conditions. CD8^+^ T cells have also been implicated due to their cytotoxic potential [91]. Under inflammatory conditions, neurons and glial cells may express MHC class I molecules, potentially enabling T cell-mediated cytotoxicity. Dopaminergic neurons are frequently discussed in this context; however, much of the supporting evidence is derived from experimental models, and its relevance to human pathology remains to be clarified [92]. Peripheral immune responses are closely linked to BBB integrity [93]. Compromised BBB function may allow infiltration of immune cells, including T lymphocytes and monocyte-derived macrophages, into the CNS. Chemokines such as CCL2 and CXCL10 are involved in this process [94], although the timing and extent of infiltration vary across experimental systems and disease conditions. Systemic inflammation has also been proposed to influence CNS pathology, although its role as a primary driver versus a secondary amplifier remains under investigation. The concept of CNS “priming” in aging or chronic inflammation has been proposed, although its cellular and molecular basis is not yet fully defined [95]. Alterations in microbiota composition may influence immune tone and T cell polarization; however, causal relationships remain under debate [96]. The hypothesis that α-Syn pathology may originate in the gut or be modulated by peripheral factors prior to CNS involvement has been proposed, although evidence remains inconclusive [97]. B cell responses, including the production of antibodies against α-Syn, have also been reported [98]. The functional significance of these antibodies—whether protective, neutral, or reflective of disease activity—remains unclear. Overall, adaptive immune mechanisms contribute to synucleinopathies in a complex and context-dependent manner. Interactions between antigen presentation, T cell activation, and peripheral immune signaling are likely to evolve over the course of disease progression. Within this context, LPLs may intersect with adaptive immune processes as modulators rather than primary drivers. Through their effects on membrane organization and lipid microdomains, they may influence antigen presentation and immune cell interactions. However, supporting evidence remains limited and largely indirect, and further studies are required to clarify these mechanisms.

### 3.6. Oxidative Stress and Mitochondrial Dysfunction

LPLs may influence mitochondrial function through effects on membrane properties and lipid-mediated signaling pathways. Redox imbalance and mitochondrial dysfunction are closely associated in synucleinopathies, although their causal relationship remains incompletely defined [99]. In some contexts, distinguishing cause from consequence may be difficult. Dopaminergic neurons are often considered especially vulnerable, primarily due to their high metabolic demand and the intrinsic reactivity of dopamine [100]. However, the relative contribution of these factors remains to be clarified and may vary depending on the experimental system. Reactive oxygen and nitrogen species accumulate under these conditions, arising from multiple cellular sources rather than a single origin. Mitochondria are frequently emphasized, particularly when electron transport chain function is impaired [101]. Defects in complex I have been reported and may promote electron leakage [102], although findings across studies are not fully consistent. Microglia also contribute to reactive species production through NADPH oxidase and inducible nitric oxide synthase (iNOS) [103]. In addition, dopamine metabolism generates reactive intermediates that further contribute to oxidative stress [104]. These processes are often described as distinct but are likely to occur concurrently within affected cells and tissues. Mitochondrial dysfunction is closely linked to these processes, although the directionality of these interactions remains unclear. α-Syn has been reported to associate with mitochondrial membranes [105] and may interfere with protein import machinery such as TOM20 [106]. Alterations in mitochondrial dynamics, including increased fragmentation mediated by proteins such as Drp1, have also been reported [107]. The temporal relationship between these changes and disease progression remains to be determined. Mitophagy impairment, particularly involving the PINK1–Parkin pathway, has been described [108]. This may lead to the accumulation of damaged mitochondria, although whether this represents a primary defect or a secondary consequence is still under investigation. Mitochondrial damage can also result in the release of mitochondrial DNA and other signals that further modulate inflammatory responses [109]. Oxidative stress can induce post-translational modifications of α-Syn, including oxidation and nitration, which are associated with increased aggregation propensity [38]. These interactions have been proposed to form feedback mechanisms, although their strength appears to vary depending on experimental conditions. Importantly, these processes are not restricted to neurons. Astrocytes may exhibit reduced antioxidant capacity [110], and glial cells also contribute to redox dysregulation [111], whereas in microglia, redox signaling can sustain inflammatory activation [112]. These processes are likely to be interconnected rather than independent. Energy metabolism is also affected, as reduced ATP production disrupts ion homeostasis and synaptic function [113]. Neurons are particularly sensitive to these changes, although disease progression is typically gradual. Several therapeutic approaches targeting these mechanisms have been explored. These include mitochondrial-targeted antioxidants and activation of pathways such as Nrf2 [114]. Strategies to enhance mitophagy or stabilize mitochondrial function have also been investigated [115]. Although promising in experimental models, their clinical translation remains limited. Within this context, LPLs may intersect with mitochondrial dysfunction through their effects on membrane organization and mitochondria-associated membranes, potentially influencing the interplay between redox imbalance and protein aggregation. However, supporting evidence for these interactions remains limited and largely indirect. Overall, these mechanisms are best understood as a dynamic and interconnected network rather than a single linear pathway, within which LPLs may act as modulators rather than primary drivers. The relative contribution of individual components is likely to vary depending on cellular context and disease stage.

### 3.7. Gut–Brain Axis and Systemic Inflammation

**Figure 3:**
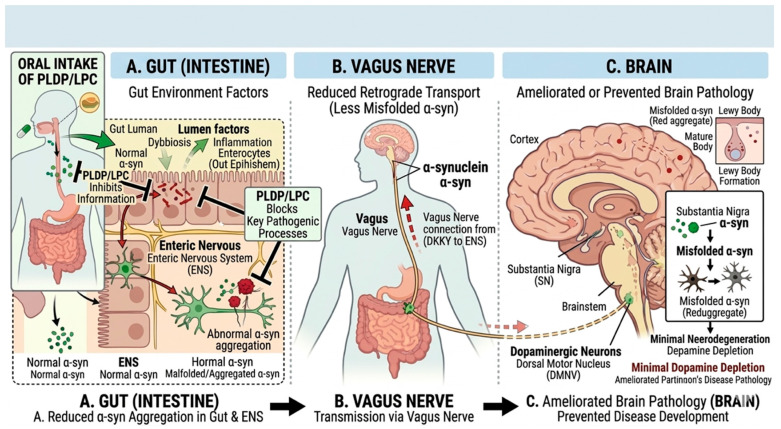
Proposed mechanism of oral PLDP/LPC in modulating the gut-to-brain axis in synucleinopathies (**A**) Oral PLDP/LPC is proposed to exert modulatory effects in the gut, potentially reducing intestinal inflammation and α-Syn aggregation within the enteric nervous system in the context of dysbiosis. (**B**) These effects may be associated with a reduction in α-Syn misfolding and its potential propagation along the vagus nerve toward the central nervous system. (**C**) Consequently, PLDP/LPC may attenuate the spread of pathology to vulnerable brain regions such as the substantia nigra, although the extent and causality of these effects remain to be fully established.

LPLs may contribute to gut–brain interactions through their roles in systemic lipid signaling and immune modulation. The gut–brain axis has been proposed as a key interface linking peripheral and central processes in synucleinopathies, although the directionality of these interactions remains incompletely defined [116]. Communication between the gastrointestinal tract and the central nervous system (CNS) is bidirectional; however, the temporal sequence and primary sites of initiation remain uncertain. One hypothesis is that the enteric nervous system may represent an early site of α-Syn pathology. Misfolded α-Syn has been detected in enteric neurons, and local inflammatory and oxidative conditions in the gut may promote its aggregation [117]. This has led to the proposal that pathological α-Syn species may propagate along the vagus nerve toward the brainstem [118]. Although supported by experimental and clinical observations, this model may not fully account for all disease presentations. The intestinal environment is highly dynamic and influenced by multiple factors, including diet, microbial metabolites, medications, and environmental exposures [119]. Under certain conditions, disruption of epithelial barrier integrity may increase intestinal permeability. This can facilitate the translocation of bacterial components such as lipopolysaccharide into the circulation, thereby promoting systemic immune activation [37]. These effects may extend beyond local tissues and contribute to systemic inflammatory responses, although their magnitude and frequency likely vary across conditions. Alterations in gut microbiota composition represent an additional factor influencing host physiology. Microbial metabolites, including short-chain fatty acids, have been shown to modulate immune function and microglial activity [118]. However, reported effects are not entirely consistent and may depend on experimental context and disease stage [119]. Peripheral immune signals can influence CNS function through multiple pathways. Lipid-mediated mechanisms, including those involving LPLs, may contribute to these interactions by linking systemic metabolic signals with immune and endothelial responses. Circulating cytokines may act on endothelial cells or signal across the BBB, and compromised BBB integrity may permit the entry of immune cells and soluble factors into the CNS [120]. These processes are likely dynamic and influenced by disease stage, systemic inflammatory status, and individual variability. Once within the CNS, these signals interact with glial cells and may promote sustained inflammatory responses. Peripheral immune activation has also been proposed to influence α-Syn pathology. Exposure of immune cells to misfolded α-Syn in the periphery may alter their activation state, potentially modifying their responses upon entry into the CNS [121]. Systemic inflammation is frequently accompanied by oxidative stress and alterations in lipid metabolism, which may in turn affect protein homeostasis [122]. The hierarchical relationships among these processes remain to be fully elucidated. Metabolic state represents an additional integrative factor. Conditions such as aging, insulin resistance, and chronic low-grade inflammation can influence mitochondrial function, immune responsiveness, and microbiota composition. These processes are likely interconnected and may collectively contribute to increased susceptibility to neurodegeneration. From a therapeutic perspective, the gut–brain axis provides multiple potential points of intervention. Strategies such as modulation of microbiota composition, restoration of barrier integrity, and attenuation of systemic inflammation have been proposed [123]. However, the relative contribution of each level and their combinatorial effects remain to be determined. Within this framework, LPLs may function as modulators linking peripheral metabolism and CNS processes. Circulating species such as lysophosphatidylcholine may influence, and potentially cross, the BBB via transport mechanisms including MFSD2A [13]. Gut microbiota may also influence systemic lipid metabolism, suggesting bidirectional interactions. Based on these observations, lipid fractions derived from PLDP, including LPC, may act at multiple levels along the gut–brain axis (see Figure 3). These effects may include modulation of intestinal conditions influencing α-Syn aggregation, regulation of systemic inflammatory tone, and indirect effects on neuronal vulnerability. However, these mechanisms remain largely speculative and require further experimental validation. Overall, the gut–brain axis is best conceptualized as a dynamic network of interacting processes rather than a single linear pathway, within which LPLs may act as modulators linking peripheral and central mechanisms. However, their precise roles remain to be defined and require further validation.

### 3.8. Integrated Pathophysiological Network and Therapeutic Implications

LPL-related pathways represent one component of lipid dysregulation in synucleinopathies and are increasingly recognized as part of a broader network that includes protein aggregation, immune activation, and metabolic disturbance [124]. Although these processes are frequently discussed together, their mechanistic integration remains incompletely understood. The extent to which these pathways are interdependent remains an open question. Rather than a single linear cascade, synucleinopathies are best conceptualized as a set of parallel and interacting processes [125], including protein misfolding, inflammatory signaling, mitochondrial dysfunction, and systemic influences. Different experimental models emphasize distinct aspects of this network. In many systems, misfolded α-Syn accumulates and interferes with intracellular trafficking and organelle function. Mitochondrial dysfunction can increase oxidative stress, which may in turn promote further protein misfolding [126]. These interactions have been described as self-reinforcing; however, their magnitude appears to vary across experimental conditions. Glial cells play central but heterogeneous roles in this context. Microglia respond to damage-associated signals by adopting pro-inflammatory phenotypes, while astrocytes exhibit alterations in metabolic and homeostatic functions [127]. These processes are often represented as reinforcing loops, although their interactions are highly context-dependent. The neurovascular unit represents an additional layer of complexity. Endothelial dysfunction and altered blood–brain barrier properties may modify communication between the CNS and peripheral circulation [128]. However, the timing and extent of these alterations vary across studies. Peripheral factors further contribute to this complexity. Within this context, LPL-related signaling may provide a link between peripheral metabolic states and central processes, although the underlying mechanisms remain incompletely understood. Systemic inflammation, metabolic disorders, and gut-derived signals have all been implicated [129], although their relative contribution to disease progression remains under investigation. In many cases, these factors may function as modulators rather than primary drivers. Aging represents a major background factor influencing multiple components of this network [130], including mitochondrial function, immune responses, and cellular resilience. However, the interactions between age-related changes and disease-specific mechanisms remain to be fully defined. From a therapeutic perspective, this complexity presents significant challenges. Targeting individual pathways—such as proteostasis, mitochondrial function, or inflammation—has shown efficacy in experimental models, although results are variable and not consistently reproducible across systems. The optimal timing of intervention also remains uncertain. Lipid-related pathways, including lysophospholipid signaling, intersect with multiple components of this network. These pathways may influence membrane properties, inflammatory signaling, and indirectly, protein homeostasis. However, their role is best characterized as modulatory and context-dependent rather than universally central. Similarly, systemic approaches targeting peripheral inflammation or gut-derived signals have been proposed, although their impact on central pathology remains to be clarified. The timing of these interventions is likely to be critical, although current evidence remains limited. The lack of reliable staging biomarkers further complicates this issue. Overall, synucleinopathies are best understood as dynamic systems of interacting processes rather than single linear pathways. Within this framework, LPL-related mechanisms may act as context-dependent modulators within a shifting network, the relative contribution of which may vary depending on cellular context and disease stage. Further studies are required to define the hierarchical relationships among these processes and to clarify the role of LPLs as potential points of intervention.

## 4. Conclusions

**Figure 4:**
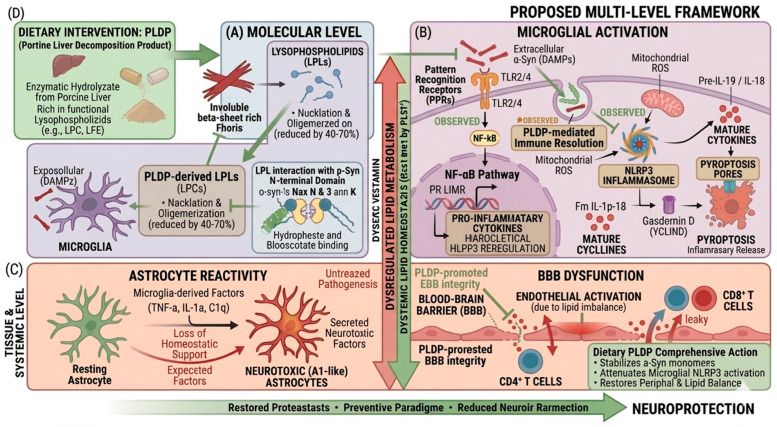
Proposed lipid-centric mechanisms in synucleinopathies: LPC-mediated modulation of proteostasis and neuroinflammation. This figure summarizes a proposed multi-level framework in synucleinopathies. (**A**) At the molecular level, α-Syn monomers may undergo aggregation into oligomeric and fibrillar species, potentially modulated by LPLs at early stages of aggregation. (**B**) At the cellular level, extracellular α-Syn may engage microglial pattern recognition receptors (e.g., TLR2/4), which is associated with NF-κB signaling, cytokine production, and potential NLRP3 inflammasome activation. (**C**) At the tissue level, neuroinflammatory responses may contribute to astrocyte activation, endothelial dysfunction, and blood–brain barrier impairment. (**D**) Lipid-based interventions, including PLDP as a source of LPLs such as LPC, may modulate both proteostatic and inflammatory pathways; however, these interactions remain hypothetical and context-dependent.

In this review, we have examined LPLs as potential points of interaction among neuroinflammatory signaling, lipid dynamics, and proteostatic processes in synucleinopathies [131,132,133]. Although LPLs are classically described as bioactive lipid mediators, accumulating evidence suggests that their functions extend beyond canonical signaling roles to include modulation of membrane organization, protein aggregation, and immune responses [41,134,135]. In particular, their reported influence on α-Syn aggregation and microglial activation positions LPLs at a potential interface between proteinopathy and inflammation. However, the extent to which these processes are mechanistically linked through LPL signaling remains incompletely defined [136,137,138,139]. Within this framework, we considered a lipid-centered perspective in which lipid dysregulation contributes to disease-related processes. Whether such changes represent initiating events or secondary adaptations is likely to depend on specific biological and experimental contexts [140,141,142]. This perspective provides a basis for re-evaluating the temporal relationships among pathological processes, although current evidence remains limited. For example, it has not yet been established whether individual LPL species directly modulate the conformational dynamics of aggregation-prone proteins such as α-Syn under physiological conditions [143]. Addressing this question will require integrated structural, biochemical, and lipidomic approaches, supported by quantitative analyses. An additional consideration is the context-dependent nature of LPL signaling. Bulk lipid measurements provide limited resolution, and future studies should emphasize spatial and temporal dynamics of lipid distribution, particularly within membrane microdomains and localized signaling environments [12,16]. The translation of these localized lipid changes into cellular and tissue-level effects remains to be fully elucidated. From a translational perspective, lipid-related pathways may represent alternative therapeutic targets for modulating disease processes. Rather than focusing on single molecular targets, strategies that influence membrane properties or broader signaling states have been proposed [142,144,145]. Multi-component systems such as PLDP have also been explored in this context, although their composition, reproducibility, and mechanisms of action remain to be clarified [140]. Early intervention has been proposed as a potentially effective strategy, particularly if lipid dysregulation contributes to initial alterations in cellular homeostasis [131]. However, this concept is primarily supported by preclinical evidence, and validation in human studies remains limited [146]. Furthermore, factors such as dosing, timing, and blood–brain barrier permeability present additional challenges for clinical translation [147]. Overall, lipid-mediated mechanisms represent one component of a broader network involving protein aggregation, inflammation, and metabolic regulation. Their relative contribution remains to be defined, and their importance may vary depending on disease stage and cellular context. Within this framework, LPLs can be considered not only as signaling molecules but also as modulators of cellular environments that may indirectly influence protein homeostasis and inflammatory responses (see Figure 4). Further studies are required to define the mechanistic basis of these interactions under both physiological and pathological conditions. While emerging evidence suggests potential roles of LPLs in synucleinopathies, current findings remain limited and largely preclinical, and further studies are required to establish their relevance to human disease.

## 5. Future Perspectives and Limitation

While the lipid-centric framework proposed here offers a novel integration of proteostasis and neuroinflammation, several limitations must be acknowledged. First, much of the evidence supporting the neuroprotective roles of LPLs and PLDP is derived from preclinical models. Given the inherent differences between animal models and human synucleinopathies, the translatability of these findings remains to be clinically validated. Second, while LPLs show potential in modulating α-synuclein aggregation and inflammatory signaling, the precise causal relationships and long-term effects of lipid-based interventions are not yet fully established. Future research should prioritize longitudinal human studies and robust replication of these mechanistic pathways to bridge the gap between experimental observations and therapeutic application.

## Data Availability

No new data were created or analyzed in this study.

## Abbreviations

α-Syn, α-synuclein; A1/A2, astrocyte phenotypes; ATX, autotaxin; BBB, blood–brain barrier; CCL2, C–C motif chemokine ligand 2; CNS, central nervous system; COX-2, cyclooxygenase-2; DAMPs, damage-associated molecular patterns; DLB, dementia with Lewy bodies; EAAT, excitatory amino acid transporter; ERK, extracellular signal-regulated kinase; ETC, electron transport chain; GPCR, G protein-coupled receptor; GSDMD, gasdermin D; HIF-1α, hypoxia-inducible factor 1α; IFN-γ, interferon gamma; IL, interleukin; iNOS, inducible nitric oxide synthase; IRF, interferon regulatory factor; JAK, Janus kinase; JNK, c-Jun N-terminal kinase; LPA, lysophosphatidic acid; LPC, lysophosphatidylcholine; LPLs, lysophospholipids; LPPs, lipid phosphate phosphatases; LPS, lysophosphatidylserine; MAPK, mitogen-activated protein kinase; MAMs, mitochondria-associated membranes; MFSD2A, major facilitator superfamily domain-containing protein 2A; MHC, major histocompatibility complex; MSA, multiple system atrophy; mTOR, mechanistic target of rapamycin; NF-κB, nuclear factor kappa B; NLRP3, NOD-like receptor family pyrin domain containing 3; NOX2, NADPH oxidase 2; PAMPs, pathogen-associated molecular patterns; PD, Parkinson’s disease; PI3K, phosphoinositide 3-kinase; PLDP, porcine liver decomposition product; PRRs, pattern recognition receptors; RAGE, receptor for advanced glycation end products; RNS, reactive nitrogen species; ROS, reactive oxygen species; S1P, sphingosine-1-phosphate; SCFAs, short-chain fatty acids; STAT, signal transducer and activator of transcription; TLR, Toll-like receptor; TNF-α, tumor necrosis factor alpha; TOM20, translocase of outer mitochondrial membrane 20; Tregs, regulatory T cells; VEGF, vascular endothelial growth factor.
